# Measuring unbearable psychache in suicide risk: psychometric evidence for the Italian UP3 and comparison with the psychache scales

**DOI:** 10.3389/fpsyg.2025.1676675

**Published:** 2025-11-04

**Authors:** Nicole Bungaro, Laura Dattilo, Marco Innamorati, Michela Balsamo, Cecilia Blandizzi, Chiara Alessio, Francesco De Vincenzo, Rossella Mattea Quinto, Federica Genova, Anna Contardi, Leonardo Carlucci

**Affiliations:** ^1^Department of Health and Life Sciences, European University of Rome, Rome, Italy; ^2^Department of Human Studies, University of Foggia, Foggia, Italy; ^3^Department of Psychology, University of Chieti-Pescara, Chieti, Italy

**Keywords:** pain, suicide, ideation, behavior, depression, assessment

## Abstract

**Introduction:**

Psychache, or mental pain, is considered an independent predictor of suicide risk. Recent perspectives highlight the unbearable aspect of psychache as an imminent trigger for suicidal crisis. To assess this specific dimension, the Unbearable Psychache Scale (UP3) was developed from the original Psychache Scale (PAS). Although preliminary studies investigated UP3’s psychometric properties, its discriminant validity in assessing suicide risk remains unclear.

**Methods:**

Thus, two cross-sectional studies were conducted on Italian community samples to examine the factorial structure, internal consistency, and scalability of the Italian UP3, and to compare its incremental and discriminant validity with PAS13/PAS10 in identifying individuals at risk for suicide.

**Results:**

Study #1 (N = 707) confirmed the UP3’s unidimensional structure, with good model fit, internal consistency, and scalability. In Study #2 (N = 257), the UP3 correlated moderately to strongly with PASs, depression, and suicide risk. ROC analyses indicated that UP3 achieved comparable accuracy to PAS13 and PAS10 for recent suicide risk (AUCs 0.75–0.83), but lower accuracy for lifetime suicidal ideation (AUC = 0.681) and behaviors (AUC = 0.777). Sensitivity analyses revealed that UP3 prioritized sensitivity over specificity, with low Positive Predictive Values across outcomes: PPV was 0.42 for recent suicidal ideation, and 0.21 for recent suicidal behaviors, compared with slightly higher but still limited values for PAS scales. Hierarchical models showed that UP3 did not demonstrate incremental validity over PAS13 in predicting either recent or lifetime suicide risk.

**Discussion:**

The UP3 appears particularly sensitive to acute unbearable psychache, performing better in detecting recent than lifetime suicide risk. Although it does not outperform PAS scales, the UP3’s brevity and focus on the intolerable component of psychache make it suitable for use alongside other measures as part of a comprehensive suicide risk evaluation in clinical or large-scale contexts.

## 1 Background

According to Shneidman (1996), mental pain (also known as psychache) is a major characteristic of the suicidal mind (Pompili, 2024). It is defined as an aversive and pervasive state of distress associated with intense and negative feelings such as guilt, fear, angst, loneliness, and helplessness (Tossani, 2013). Although some researchers (Fava et al., 2019) have considered mental pain a transdiagnostic feature of psychiatric disorders and health conditions, especially the presence of unbearable psychache, mixed with intense and complex affective states, was considered characteristic of individuals at an acute risk for suicide (Hendin et al., 2007).

The presence of mental pain has been strictly linked to severe depression (Mee et al., 2006). However, mental pain is distinguishable from distress symptoms–especially those related to suffering–that characterize depression (Pompili et al., 2022a). Indeed, several studies conducted with different populations (e.g., students, psychiatric patients, individuals with chronic pain, offenders, and the homeless) have shown that high levels of mental pain significantly predict suicide risk (Pérez-Balaguer et al., 2025; da Silva et al., 2024; Coohey et al., 2015; Levinger et al., 2016; Pereira et al., 2010), and identified psychache as a distinct risk factor for suicide (Troister and Holden, 2010; Wang et al., 2024). For example, a 2-year longitudinal study reported that psychache was the only significant predictor of suicidal ideation in a student sample (Troister and Holden, 2012). Moreover, psychache appears to mediate the relationship between hopelessness and depression with suicide risk (DeLisle and Holden, 2009; Nahaliel et al., 2014), as well as the relationship between general distress and suicidal ideation (Campos et al., 2017).

From a theoretical perspective, only when mental pain is experienced as intolerable, suicide becomes the only way to cope with one’s suffering (Soper, 2023), in line with the Cubic Model of suicide (Shneidman, 1996, 1998), which posits that suicide occurs only when three elements (i.e., psychache, pressure, and perturbation) converge at critical levels (Shneidman, 1996; Pompili, 2024). Given that mental pain is a key factor in suicide risk, it is important to determine whether it is specifically its unbearable form that increases the risk for suicide, or whether other aspects of mental pain (e.g., frequency, and intensity) could play a contributory role (Pachkowski et al., 2019).

Over the years, several instruments have been developed to assess mental pain, such as the Psychache Scale (PAS; Holden et al., 2001), the Orbach and Mikulincer Mental Pain scale (OMMP; Orbach et al., 2003), the Psychological and Physical Pain-Visual Analog Scale (PPP-VAS; Olié et al., 2010), the Mee-Bunney Psychological Pain Assessment (MBPPAS; Mee et al., 2011), the Three-Dimensional Psychological Pain Scale (TDPPS; Li et al., 2014), the Suicide Cognition Scale (SCS; Bryan et al., 2014), and the Mental Pain Questionnaire (MPQ; Fava et al., 2019). While these tools offer valuable insights into the emergence of suicide risk related to mental pain, most of them do not sufficiently capture its unbearable form, as they comprise items that assess various aspects of mental pain or focus primarily on related cognitions and experiences (Pachkowski et al., 2019).

The 13-item Psychache Scale (PAS13), developed by Holden et al. (2001) and based on Shneidman’s (1996) original conceptualization, was the first quantitative and standardized measure created specifically to assess psychache. It remains widely used and has demonstrated a strong association with suicide risk across different samples (e.g., Chin and Holden, 2013; Troister et al., 2013; Patterson and Holden, 2012). PAS13 also proved to be a valid instrument for discriminating between suicide attempters and non-attempters (Campos and Holden, 2020; Lambert et al., 2020; Holden et al., 2001). However, only three items of the PAS13 target unbearable psychache, resulting in a general measure of mental pain that is not specific to detect the intolerable form of psychache (Pachkowski et al., 2019). Moreover, although the PAS13 has demonstrated satisfactory psychometric properties, several studies have reported inconsistent findings regarding its factorial structure, supporting either a unidimensional model or a two-factor solution (e.g., Boye et al., 2024; Ordóñez-Carrasco et al., 2022; Campos et al., 2019; Troister and Holden, 2013; Holden et al., 2001). For this reason, Blandizzi et al. (2025) tested and developed a 10-item version of the Psychache Scale (PAS10) by conducting a Mokken Scale Analysis (MSA) on the original PAS13. The PAS10 showed strong internal consistency and retained a unidimensional structure, addressing previous concerns regarding the factorial instability of the PAS13. Moreover, it demonstrated strong correlations with suicidal ideation and depression, as well as good discriminative capacity for identifying individuals at a higher risk for suicide. However, despite its solid psychometric properties, the PAS10 still considers items that do not specifically focus on unbearable psychache, the same way the original scale does (Blandizzi et al., 2025).

In response to this gap, Pachkowski et al. (2019) devised the Unbearable Psychache scale (UP3), selecting three items of the PAS13, specifically designed to assess the intolerable form of psychache (Pachkowski et al., 2019). Preliminary evidence supports its strong internal consistency, predictive validity, and incremental utility in explaining suicidal ideation beyond general psychache and other suicide-related constructs (Namlı et al., 2022; Pereira and Campos, 2022; Pachkowski et al., 2019). Conversely, another study found that UP3 did not outperform PAS13 in predicting suicide risk (Campos and Holden, 2020). The inconsistent results regarding the comparison between the UP3 and the PAS in identifying individuals at risk for suicide may be attributable to differences in how suicide risk was operationalized. Particularly, these differences may depend on whether the focus is on current or lifetime risk, as well as whether ideation or suicidal behavior is being evaluated. For instance, Pachkowski et al. (2019) focused on acute suicidal ideation, whereas Campos and Holden (2020) considered past suicide attempts. Nevertheless, no studies to date have explicitly distinguished between these perspectives.

Given the growing interest in the unbearable dimension of mental pain and the relative novelty of the UP3, along with the presence of mixed findings regarding its predictive validity, further research is needed to clarify its psychometric properties and clinical utility. Specifically, more investigation is required in terms of the factorial structure, reliability, and ability to discriminate individuals at risk for suicide, especially when UP3 is compared to the longer versions of the PAS.

Thus, two studies were conducted. Study #1 aimed to: (a) examine the psychometric properties of the Italian version of the UP3, and (b) assess its unidimensionality and internal consistency; Study #2 aimed to: (c) confirm the UP3 dimensionality, and assess its measurement invariance across sex, (d) compare the UP3’s validity in discriminating levels of current and lifetime suicide risk (suicidal ideation and behavior) with the performance of the PAS13 and PAS10, and (e) explore the incremental and convergent validity of the UP3 with measures of depression and suicide risk. Given the exploratory nature of the study, no hypothesis was formulated regarding whether the UP3 has greater predictive power than the PAS. On the contrary, we hypothesized strong correlations between UP3 and PAS scores, and strong correlations between measures of mental pain and severity of depression and measures of suicide risk, although not so high as to indicate poor discrimination between these constructs (Cohen, 2013).

## 2 Study #1

### 2.1 Methods

#### 2.1.1 Participants and procedure

Participants were 709 adults (604 females and 104 males, 1 preferred not to say) recruited from the general Italian population. Mean age of the participants was 30.66 years (SD = 8.48; range = 18 to 80 years). Two participants were excluded due to missing values, resulting in a final sample of 707 participants (mean age = 30.67, SD = 8.49). Table 1 summarizes the sociodemographic characteristics of the sample.

**Table 1:** Sociodemographic characteristics of the first and second sample.

Variable	Study #1 (*N* = 707)		Study #2 (*N* = 257)	
	Number	Percentage	Number	Percentage
Sex				
Females	602	14.7	194	75.5
Males	104	85.1	60	23.3
Non binary	1	0.1	3	1.2
Age – Mean | SD	30.7	8.49	31.8	15.2
Marital status				
Single	432	61.0	159	61.9
Married/Partnered	257	36.4	86	33.5
Divorced	14	2.0	9	3.5
Widow	4	0.6	3	1.2
Job status				
Unemployed	66	9.3	11	4.3
Employed	429	60.7	88	34.2
Retired	13	1.8	19	7.4
Students	199	28.2	139	54.1
School attainment				
<8 years			3	1.2
13 years			121	47.1
16 years			45	15.5
>18 years			88	34.2
Self-reported past psychiatric diagnosis			233 (no)	90.7
			24 (yes)	9.3

Inclusion criteria were age of 18 + years and the ability to complete the online protocol. Exclusion criteria were age under 18 years and an inability to complete the assessment for any reason, including refusal of informed consent.

The sample was recruited via online groups (e.g., Facebook) and on university campuses. Researchers approached students from universities in Central and Southern Italy (i.e., Rome, Chieti) through advertisements that described the study objectives and the inclusion criterion. Students were also asked to share the information with family members and friends. Participation was voluntary, and all participants provided written informed consent. The assessment protocol was administered via Google Modules to ensure anonymity, collecting only responses to the UP3 and demographic data. No personal identifiers, such as email addresses, were recorded. Each participant received information on the study objectives, their rights under the Italian Personal Data Protection Code (D. Lgs. no. 196/2003), and emergency contact details before consenting to participate to the study. Only those who consented could access the survey, which took approximately 15 min to complete. Participants received no compensation. The study was approved by the Ethics Committee of the European University of Rome and adhered to Helsinki Declaration standards.

#### 2.1.2 Measures

*Unbearable psychache scale* (UP3). The UP3 scale was developed by Pachkowski et al. (2019), who selected three items from the original 13-item PAS scale (items 10, 11, 12). Items (#10 “I cannot take my pain any more,” #11 “Because of my pain, my situation is impossible,” and #12 “My pain is making me fall apart”) were chosen because they reflect the intolerable nature of psychache. Each item is rated on 5-point Likert scale (from 1 = “strongly disagree” to 5 = “strongly agree”). Higher scores are associated with more severe unbearable psychache.

#### 2.1.3 Statistical analysis

To support the scale’s unidimensionality and account for the ordinal response format, a Confirmatory Factor Analysis (CFA) using a Diagonally Weighted Least Squares (DWLS) estimator on a polychoric correlation matrix was performed. A congeneric three-item one factor CFA (the minimum required to ensure a stable and reliable measurement model) represents a just-identified model, meaning it has zero degrees of freedom and its estimation could not provide meaningful results on model fit results. Most CFA specifications involve congeneric indicators presumed to measure the same construct with variable factor loadings and independent measurement errors (Brown, 2015). When this conditions could not be reached, assuming a more restrictive tau-equivalent model represent a viable solution to cope for the brink of being under-identified three-item model (Graham, 2006). Therefore, a tau-equivalent model was tested (constraining loadings equally), which increases degrees of freedom and allows for non-zero degrees of freedom and the estimation of model fit indices. This model is also theoretically justifiable, as the UP3 was designed to assess a single construct, with items reflecting equivalent facets of the latent trait (Pachkowski et al., 2019). Model fit was evaluated using the chi-squared test, Comparative Fit Index (CFI), Tucker Lewis Index (TLI), Root Mean Square Error of Approximation (RMSEA), and Standardized Root Mean Square Residual (SRMR), with nonsignificant chi-squared (*p* > 0.05), CFI and TLI ≥ 0.90, RMSEA and SRMR ≤ 0.08 indicating adequate fit (Schermelleh-Engel et al., 2003). CFA was performed using Lavaan package for R.

Subsequently, a Mokken scale analysis (MSA) was conducted to evaluate UP3 measurement properties, and to scale participants and items along the latent trait (Sijtsma and van der Ark, 2017). Compared to the parametric variants of Item Response Theory (IRT), MSA does not require strict assumptions about the shape of data distribution (Balsamo et al., 2020). Both monotone homogeneity (i.e., responders and items can be ordered along a common latent trait, items are locally independent, and items response functions are monotonically non-decreasing) and double monotonicity (items can also be ordered invariantly) were considered, following Sijtsma and van der Ark (2017). The monotone homogeneity model is sufficient for scales that order participants along one dimension. MSA was performed with the Mokken package for R.

Local dependency was assessed by Straat et al. (2016) procedure, based on W1, W2, and W3 indices, which allowed to assess the presence of local independence for the UP3 items before performing the Automated Item Selection Procedure (AISP). A vector of scaling criteria (0.2 to 0.7 in steps of 0.05) was used to evaluate scalability. From the AISP results, item-pair scalability coefficients (Hij), item scalability coefficients (Hi), scale scalability coefficient (H), and the H_T_ coefficient were computed to express the accuracy of item ordering (Loevinger, 1948; Ligtvoet et al., 2010), with satisfactory values defined as Hij > 0, Hi and H ≥ 0.3, and H_T_ ≥ 0.3. Monotonicity was assessed separately using Mokken package procedures.

### 2.2 Results

A tau-equivalent one-factor model showed the following fit indices: *χ*^2^_3_ = 2.244, CFI = 1.000, GFI = 0.999, SRMR = 0.030, RMSEA = 0.013 (95% CI RMSEA = 0.011–0.077). The model fit indices are consistent with a good fit of a tau-equivalent model.

No local dependency was observed between UP3’s items. All H_ij_ values were positive and above 0.3; Hi indices also exceeded 0.3 (ranging between 0.80 and 0.86), and H was 0.82 (see Table 2). The H^T^ index was acceptable (H^T^ = 0.41), indicating acceptable item ordering. In addition to this, reliability indices indicated high internal consistency (Cronbach’s alpha = 0.905; LCRC = 0.906). Overall, the UP3 satisfied the criteria for the monotone homogeneity model and was retained for subsequent analyses.

**Table 2:** Reliability, Mokken scalability coefficients, and CFA loadings of UP3 items and scale across first and second sample.

	Study #1 (*N* = 707)				Study #2 (*N* = 257)			
	Mokken			CFA	Mokken			CFA
Item	Scale	Hi	SE	Std λ	Scale	Hi	SE	Std λ
UP_1	1	0.800	0.020	0.855	1	0.877	0.024	0.867
UP_2	1	0.857	0.013	0.910	2	0.892	0.021	0.973
UP_3	1	0.817	0.016	855	2	0.869	0.023	0.876
H (SE)	0.824 (0.016)				0.879 (0.021)			
H^T^	0.409				0.323			
MS	0.913				0.935			
*α*	0.905				0.929			
LCRC	0.906				0.929			

Hij, item scalability coefficient; SE, Standard Error of item scalability; MS, Molenaar-Sijtsma method; *α*, Cronbach’s alpha; LCRC, Latent Class Reliability Coefficient.

CFA standardized loadings and MSA descriptive data on UP3 are presented in Table 2.

## 3 Study #2

### 3.1 Methods

#### 3.1.1 Participants and procedure

For Study #2, 257 adults (194 females and 60 males, 3 preferred not to say) were nonrandomly recruited from the general Italian population. The mean age of the participants was 31.81 years (SD = 15.24; range = 18 to 76 years). Inclusion and exclusion criteria, and procedures as detailed in Study #1. Sociodemographic characteristics of the sample are provided in Table 1.

Participants were administered a checklist assessing sociodemographic variables and a battery of psychological questionnaires, which included UP3 (Pachkowski et al., 2019), PAS13 (Holden et al., 2001), Suicidal History Self-Rating Screening Scale (SHSS; Innamorati et al., 2011), and Patient Health Questionnaire Depression Scale (PHQ–9; Kroenke et al., 2001). PAS13 items shared with the UP3 were administered twice. UP3 was included at the beginning of the protocol, and PAS13 at the end of the protocol.

#### 3.1.2 Measures

*Unbearable Psychache Scale* (UP3; Pachkowski et al., 2019) was described in Study #1.

*Psychache Scale* (PAS13; Holden et al., 2001). The PAS is a 13-item self-report scale evaluating the presence and frequency of psychological pain and how well the respondent can tolerate the level of pain they are experiencing. The scale provides two response rate options. The first nine items are rated on a 5-point Likert-type scale, from “never” to “always.” The last four items use a different 5-point Likert-type scale, from “strongly disagree” to “strongly agree.” The PAS has demonstrated satisfactory reliability and validity (Holden et al., 2001; Mills et al., 2005; Pompili et al., 2022a; Pompili et al., 2022b).

*Psychache Scale – ten items* (PAS10; Blandizzi et al., 2025). The PAS10 derives from the work of Blandizzi et al. (2025), who examined the psychometric properties of the 13-item PAS scale and concluded that three items (item #6, #8, #12) should be removed. For the present study, the 10 items identified for the PAS10 were extracted from the administration of the full PAS13 (described above).

*Suicidal History Self-Rating Screening Scale* (SHSS; Innamorati et al., 2011). The SHSS is a 16 + 16-item measure assessing death thoughts, suicidal ideation (i.e., death wishes, active suicidal ideation) and behavior (i.e., suicide attempts, interrupted or self-interrupted attempts, and preparatory acts) in the last 12 months (SHSS-C) and lifetime except for the last 12 months (SHSS-L). Participants should respond to items on a 4-level Likert-type scale ranged from 0 = “Never,” to 3 = “Always.” This module was derived from the original 18-item version, which measures the lifetime and last 12 months suicide risk and exhibited satisfactory reliability and validity in past studies (Innamorati et al., 2011). In the present sample, Cronbach’s alphas were 0.95 and 0.92, respectively for the SHSS-L and for the SHSS-C.

*Patient Health Questionnaire Depression Scale* (PHQ–9; Kroenke et al., 2001), is a 9-item measure that evaluate whether depressed symptoms have been present over the last 2 weeks. People rate items on 4-point Likert scales that ranged from 0 = “Not at all,” to 4 = “Very much.” Higher scores indicate more severe depressive symptoms. The cumulative score, ranging from 0 to 27, with scores ≥ 10 suggesting moderate to severe depression. Cronbach’s alpha in the present study was 0.86.

#### 3.1.3 Statistical analysis

Confirmatory Factor Analysis (CFA) using Diagonally Weighted Least Squares (DWLS) estimator was performed in order to support UP3’s unidimensionality as defined in Study #1. A Mokken model and procedures suggested by Straat et al. (2016) were performed. Goodness-of-fit indices, as well as internal consistency indices used are indicated in Study #1. In addition, a *multiple indicators, multiple causes* (MIMIC) model was tested to assess UP3 measurement invariance across sex. In MIMIC model both the latent factors and indicators are regressed onto a dummy variable that denotes group membership (sex in our case). A significant direct effect of the dummy code (covariate) on the latent factor indicates population heterogeneity (group differences on latent means). MIMIC models are more parsimonious and require smaller sample sizes than multiple-groups CFA, analyzing a single measurement model and input matrix (Brown, 2015).

Receiver Operating Characteristic (ROC) curves with the maximize Youden’s index method and Spline smoothing were used to assess ability of scores of the three PAS versions (PAS13, PAS10, UP3) in discriminating participants with higher suicidal ideation and behavior, and defining cut-off scores with satisfactory sensitivity and specificity.

Hence, two classification variables were created by summing items from the SHSS-C and SHSS-L. Suicidal ideation was assessed with four items [i.e., active suicidal ideation (#4), suicidal ideation with method (#5), suicidal ideation with planning (#6), suicidal ideation with intent to act (#9)]. Suicidal behavior was assessed with three items [i.e., aborted attempt (#10), preparatory act (#11), and actual attempt (#12)]. Participants scoring at or above the 75^th^ percentile were classified as cases, with the threshold applied separately for suicidal ideation and behavior when the two endpoints were analyzed independently; whereas participants below this threshold were classified as controls. These items were selected because they closely correspond to the indicators included in the Columbia-Suicide Severity Rating Scale (C-SSRS) and represent the suicidal phenomena most frequently identified in the literature (Posner et al., 2011).

We reported the Area Under the Curve (AUC) as a measure of scores’ ability to categorize individuals, its post-hoc power test computation (two-sided and *p* < 0.05), and the relative effect size (ES). AUC is interpreted as the probability that a randomly sampled respondent is correctly assigned to the appropriate group (Hanley and McNeil, 1982). AUC directly represents the overall accuracy of the measures, with scores ranging between 0 and 1. AUC ≥ 0.9 indicates excellent predictive accuracy, scores between 0.8 and 0.9 a good accuracy, between 0.7 and 0.8 fair accuracy, and ≤0.7 poor accuracies (Metz, 1978; Somoza and Mossman, 1992). AUC can also be used as a general effect size measure (Smithson, 2025); nevertheless, since a single agreed-upon definition of ES is not present, we converted AUC values into Cohen’s *d* using the Salgado (2018) tables. Sensitivity, specificity, Positive Predictive Value (PPV), and Negative Predictive Value (NPV) were also reported for PAS scores maximizing Youden’s index.

In addition, to verify the existence of a statistical difference between the ROC curves based on the different PAS versions, a series of pairwise comparisons were performed (UP3 vs. PAS13, UP3 vs. PAS10), using DeLong’s test for correlated AUCs (DeLong et al., 1988). For each comparison, we calculated ΔAUC along with 95% Confidence Intervals (CIs) to provide a formal assessment of the difference in discriminative performance. Since the PAS10 is nested within the PAS13, we do not compute any comparison test among them (e.g., pairwise comparison).

Two hierarchical logistic regressions with SHSS-C and SHSS-L as criteria were performed to analyze the incremental validity of UP3 beyond PAS13. In each model, PAS13 was included as the first variable (Model #1); subsequently, UP3 was added to PAS13 in Model #2. Variance Inflation Factor (VIF) and Tolerance were assessed to detect multicollinearity. VIF measures how much the variance of a regression coefficient is inflated due to multicollinearity; VIF > 10 indicates significant multicollinearity. Tolerance is the inverse of VIF, so tolerance < 0.1 signals multicollinearity. Model’s overall fit was assessed by pseudo Nagelkerke *R*^2^, *χ*^2^ value, the change in the model’s explanatory power (*χ*^2^ omnibus test) and its *p*-value. Estimates, Wald’s *Z*, Odds Ratio (OR), and its 95% CI, Standard Error (SE) for each model/predictor were also reported.

Inter-correlations and Paired Correlations between the three PASs versions and the PHQ-9 and SHSS scores were analyzed using Zou’s Confidence Intervals (Zou, 2007). Confidence intervals for the difference between the *Z*-transformed correlation coefficients including zero suggest that correlations are not significantly different. All statistical analyses were performed with the packages lavaan, Mokken, pROC, cocor and jmv r package in R.

### 3.2 Results

#### 3.2.1 Confirmatory factor analysis, Mokken scale analysis, and MIMIC model

CFA on the second sample demonstrated a good fit of the tau-equivalence model to the data (*χ*^2^_3_ = 0.166, *p* = 0.92; RMSEA = 0.002 [95% CI = 0.001/0.044]; SRMR = 0.014; CFI = 1; GFI = 1), consistent with the assumption of a single latent factor underlying the three items (see Table 2). MSA descriptive statistics for the UP3 items and total scale replicated those of Study #1, showing adequate item scalability (Hi = 0.869–0.892), overall scale scalability (H = 0.879), and internal consistency (*α* = 0.929; LCRC = 0.929), consistent with a unidimensional structure (see Table 2).

The MIMIC model provides a good fit to the data (*χ*^2^ (4) = 5.208, *p* = 0.21, SRMR = 0.020, RMSEA = 0.044 [90% CI = 0.001 to 0.112], TLI = 0.984, CFI = 0.989). Inclusion of the sex covariate did not alter the factor structure or produce salient areas of strain in the solution (all modification indices < 4.0). The path of Sex on UP3 was statistically significant (z = −2.759, *p* < 0.01), so that UP3 mean for females is 0.386 units higher than for their male peers.

#### 3.2.2 ROC curve analyses and pairwise comparison on SHSS-C (recent suicide risk)

ROC curves analyses were carried out to investigate the ability of the three scales in discriminating individuals with eventual presence of recent suicidal ideation or behaviors. Results demonstrated adequate to high discrimination for all scales, with broadly similar performance and a statistical power >0.90 (see Table 3).

**Table 3:** Area Under the Curve (AUC) of the Receiver Operating Characteristic Curve (ROC) Analyses for the different version of the PAS (PAS13-PAS10-UP3), and comparison of independent ROC curves.

SHSS-C	Control/case	AUC	SE	(95% CI)	Sensitivity/Specificity	Cut-off	PPV/NPV	Model	∆AUC	SE	(95% CI)	z	*p*
Subscale – cut-off													
Ideation	205/52												
13-item PAS		0.786	0.035	0.717/0.856	0.558/0.883	>17.5	0.547/0.887						
10-item PAS		0.787	0.035	0.719/0.856	0.635/0.785	>9.5	0.429/0.894	PAS13 vs. UP3	0.035	0.024	−0.012/0.082	14.434	0.148
3-item UP3		0.751	0.039	. 675/0.828	0.577/0.800	>3.5	0.423/0.882	UP3 vs. PAS10	0.036	0.024	−0.012/0.084	14.511	0.146
Behavior	236/21												
13-item PAS		0.829	0.044	0.743/0.915	0.810/0.742	>11.5	0.218/0.978						
10-item PAS		0.832	0.042	0.750/0.914	0.714/0.792	>11.5	0.234/0.969	PAS13 vs. UP3	0.007	0.032	−0.055/0.069	0.227	0.820
3-item UP3		0.822	0.048	0.729/0.915	0.714/0.763	>3.5	0.211/0.968	PAS10 vs. UP3	0.009	0.032	−0.049/0.069	0.324	0.069

SHSS-L	Control/case	AUC	SE	(95% CI)	Sensitivity/Specificity	Cut-off	PPV/NPV	Model	∆AUC	SE	(95% CI)	z	*p*
Subscale – cut-off													
Ideation	200/57												
13-item PAS		0.769	0.037	0.696/0.841	0.632/0.810	>12.5	0.486/0.885						
10-item PAS		0.773	0.037	0.701/0.844	0.719/0.730	>7.5	0.432/0.901	PAS13 vs. UP3	0.088	0.025	0.036/0.137	3.375	0.001
3-item UP3		0.681	0.042	0.600/0.763	0.316/0.945	>6.7	0.621/0.829	PAS10 vs. UP3	0.092	0.021	0.040/0.142	3.496	0.001
Behavior	223/34												
13-item PAS		0.833	0.038	0.759/0.907	0.824/0.753	>10.5	0.337/0.966						
10-item PAS		0.834	0.037	762/0.906	0.853/0.704	>7.5	0.305/0.969	PAS13 vs. UP3	0.056	0.031	−0.004/0.116	18.118	0.070
3-item UP3		0.777	0.040	0.699/0.856	0.941/0.484	>0.1	0.218/0.982	PAS10 vs. UP3	0.057	0.030	−0.002/0.115	18.931	0.058

When discriminating individuals with different suicide ideation severity (205 controls/52 cases), PAS13 yielded an AUC of 0.786 (SE = 0.035; 95% CI = 0.717/0.856; *d*ES of 1.121); PAS10 an AUC of 0.787 (SE = 0.035; 95% CI = 0.719/0.856; *d*ES of 1.126); and UP3 an AUC of 0.751 (SE = 0.039; 95% CI = 0.675/0.828; *d*ES = 0.958). Overall, performance was broadly comparable across scales: PAS13/PAS10 tended to show slightly higher and more stable sensitivity/specificity across cut-offs compared with UP3; while PPVs were generally low (0.423–0.547), and NPVs consistently high (0.882–0.894) across all scales, indicating their relative strength in ruling out risk. Overall, no scale showed a clear superiority. Additional details are provided in Table 4. Pairwise comparisons among the scales were not significant (*p* > 0.05) with a ΔAUCs between 0.035 (UP3vsPAS13), and 0.036 (UP3vsPAS10), with confidence intervals including zero and a statistical power of 0.49–0.51.

**Table 4:** Incremental validity of the UP3 over PAS13 on SHSS-C and SHSS-L.

		SHSS-C					
		Est.	SE	OR	OR 95% CI		Wald _Z_
Model#1	Constant	−20.165	0.2329	0.133	0.0843	0.210	−866**
	PAS	0.0933	0.0146	1.098	1.066	1.130	6.39**
Model#2	Constant	−20.236	0.2341	0.132	0.0835	0.209	−8.645**
	PAS	0.0868	0.0245	1.091	1.039	1.144	3.549**
	UP3	0.0297	0.0905	1.030	0.8626	1.230	0.328
		Model#1	Model#2				
*R*^2^ Nagelkerke		0.263	0.264				
*χ*^2^_(df)_		51.7 (1)**	51.8 (2)**				
Omnibus test *χ*^2^_(df)_		0.107 (1); *p* = 0.744					

		SHSS-L					
		Est.	SE	OR	OR 95% CI		Wald _Z_
Model#1	Constant	−18.188	0.2210	0.162	0.105	0.250	−8.23**
	PAS	0.0927	0.0147	1.097	1.066	1.129	6.32**
Model#2	Constant	−1.804	0.2221	0.165	0.107	0.254	−8.12**
	PAS	0.119	0.0258	1.126	1.071	1.185	4.61**
	UP3	−0.118	0.0924	0.889	0.742	1.065	−1.28
		Model#1	Model#2				
*R*^2^ Nagelkerke		0.257	0.264				
*χ*^2^_(df)_		51.6 (1)**	53.3 (2)**				
Omnibus test *χ*^2^_(df)_		1.68 (1), *p* = 0.195					

**p < 0.001.

When discriminating individuals with the presence of any recent suicidal behaviors (236 controls/21 cases), all three scales showed broadly comparable discrimination. AUCs were 0.829 for the PAS13 (SE = 0.044; 95% CI = 0.743/0.915; *d*ES = 1.344); 0.832 for the PAS10 (SE = 0.042; 95% CI = 0.750/0.914; *d*ES = 1.361); and 0.822 for the UP3 (SE = 0.048; 95% CI = 0.729/0.915; *d*ES = 1.305). Overall, sensitivity ranged approximately from 0.714 to 0.810 and specificity from 0.742 to 0.792 across cut-offs. PPVs were generally low (0.211–0.234) and NPVs consistently high (0.969–0.978), reflecting the low base rates of suicidal behaviors and indicating that all the scales are more effective in ruling out risk than in predicting positive cases. Pairwise comparisons were not significant (*p* > 0.05), with ΔAUCs ranging from 0.007 (UP3vsPAS13) to 0.032 (UP3vsPAS10), confidence intervals including zero, and low statistical power (0.08–0.16). Additional details are provided in Table 3.

#### 3.2.3 ROC curve analyses and pairwise comparison on SHSS-L (past suicide risk)

Across all lifetime indices of suicidal ideation and behaviors, PAS13 and PAS10 generally demonstrated comparable discrimination with an optimal statistical power >0.90; whereas UP3 showed lower performance for the severity of lifetime ideation, with pairwise comparisons reaching statistical significance.

When discriminating individuals with different suicide ideation severity (200 controls/57 cases), AUCs were 0.769 for PAS13 (SE = 0.037; 95% CI = 0.696/0.841; *d*ES = 1.040), and 0.773 for PAS10 (SE = 0.037; 95% CI = 0.701/0.844; *d*ES = 1.059), whereas UP3 yielded a consistently lower AUC (0.682; SE = 0.042; 95% CI = 0.600/0.763; *d*ES = 0.669). Overall, sensitivity ranged approximately from 0.316 to 0.719, while specificity from 0.730 to 0.945. PPVs were generally low (0.432–0.621) and NPVs consistently high (0.829–0.901), indicating that the scales are more effective in ruling out risk than predicting positive cases (see Table 3 for details). UP3 tended to show higher sensitivity but lower specificity, whereas PAS13 and PAS10 showed more balanced sensitivity and specificity. ROCs pairwise comparison were found to be significant (*p* < 0.001) with a statistical power >0.90, in which UP3 consistently obtained worse results. In detail, ΔAUCs were 0.088 for UP3vsPAS13 (SE = 0.025; 95% CI = 0.036/0.137; *z* = 3.375); and 0.092 for UP3vsPAS10 (SE = 0.021; 95 % CI = 0.040/0.142; *z* = 3.496).

When discriminating individuals with any lifetime suicide behaviors (223 controls/34 cases), slightly similar trend but non-significant patterns were evident. PAS13 and PAS10 performed comparably: AUCs were 0.833 (SE = 0.038; 95% CI = 0.759/0.907; *d*ES = 1.683) and 0.834 (SE = 0.037; 95% CI = 0.762/0.906; *d*ES = 1.372), respectively for PS13 and PAS10. The UP3’s AUC was moderately lower (0.777; SE = 0.040; 95% CI = 0.699/0.856; *d*ES = 1.078). Overall, sensitivity ranged approximately from 0.824 to 0.941, and specificity from 0.484 to 0.753. PPVs were generally low (0.218–0.337) and NPVs consistently high (0.966–0.982), reflecting the low base rates of lifetime suicidal behaviors. UP3 tended to show higher sensitivity but lower specificity, whereas PAS13 and PAS10 showed more balanced sensitivity and specificity. This pattern indicates that all scales are more effective in ruling out risk than in predicting positive cases. Pairwise comparisons were slightly above the threshold of statistical significance (*p* = 0.058/0.070) with ΔAUCs between 0.056 (UP3vsPAS13) and 0.057 (UP3vsPAS10), with confidence intervals including zero and a low statistical power of 0.51–0.58. Additional details are provided in Table 3.

**Figure 1:**
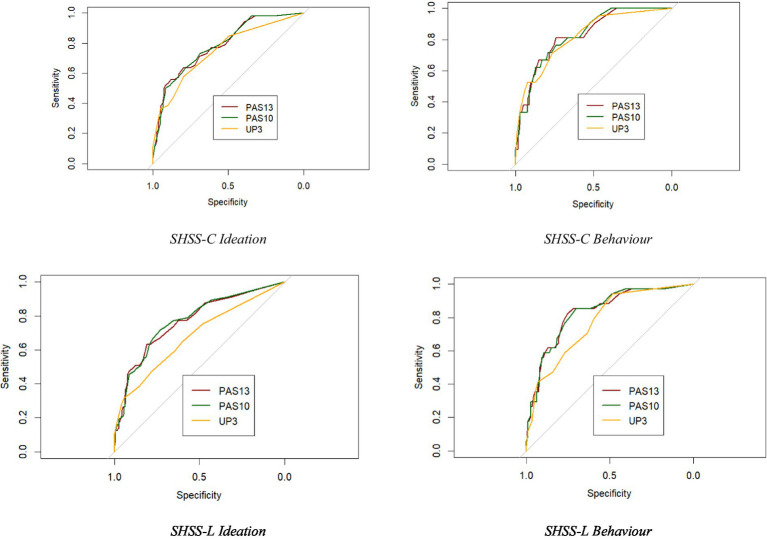
Paired ROC curves for the SHSS-C and SHSS-L suicidal ideation and behavior.

Taken together, these results indicate that while UP3 provides a very brief screener with high sensitivity, PAS13 and PAS10 show superior balance between sensitivity and specificity, yielding more robust classification across suicidality indices (see Figure 1).

#### 3.2.4 Incremental and convergent validity

Two hierarchical logistic regressions were performed to determine the incremental validity of the UP3 in predicting recent (SHSS-C) and lifetime (SHSS-L) suicide risk, accounting for PAS13 scores (see Table 4). PAS13 explained 26.3% of the variance of recent suicide risk (ORs between 1.09 and 1.098, *p* < 0.001; Negelkerke *R*^2^ = 0.263, *χ*^2^_(df)_ = 51.7_(1)_ *p* < 0.001). The UP3 (OR = 1.030, *p* = 0.743) did not contribute significantly when included in the model (*χ*^2^_(df)_ change = 0.107(1), *p* = 0.744).

PAS13 explained 26.3% of the variance of lifetime suicide risk (ORs between 1.097 and 1.126, *p* < 0.001; Negelkerke *R*^2^ = 0.257, *χ*^2^_(df)_ = 51.6_(1)_, *p* < 0.001). The UP3 (OR = 0.889, *p* = 0.202) did not contribute significantly when included in the model (*χ*^2^_(df)_ change = 1.68_(1)_, *p* = 0.195).

Inter-correlation coefficients between PAS13/PAS10 and UP3 scores were statistically significant (see Table 5). PAS13 scores correlated almost perfectly (*r* = 0.99, *p* < 0.001) with PAS10 scores, and UP3 scores correlated strongly with both PAS13 (*r* = 0.83; *p* < 0.001) and PAS10 (*r* = 0.83; *p* < 0.001) scores. To formally test whether UP3’s correlations with external measures differed from those of PAS longer versions, Zou’s (2007) method for comparing correlated correlations was applied. Specifically, we compared overlapping correlations. Δr values and 95% CIs from Zou’s procedure are reported in Table 5. In general, correlations between UP3 and external measures were lower than those of PAS longer versions. Differences were largest for lifetime suicide risk (SHSS-L: Δr = −0.078 to −0.085), when partialling out depression severity (*r* between 0.31 and 0.32; *p* < 0.001).

**Table 5:** Correlation analysis among PAS13, PAS10, UP3 and the PHQ, the SHSS-C and SHSS-L and Zou’s CI values for the Study #2.

Test total scores	PAS13	UP3	Δr	Zou’s CI	PAS10	UP3	Δr	Zou’s CI
	*r*				*r*			
PHQ-9	0.74	0.63	−0.108	−0.165/−0.058	0.73	0.63	−0.101	−0.157/−0.051
SHSS-C	0.53	0.52	−0.011	−0.073/0.049	0.53	0.52	0.011	−0.071/ 0.049
SHSS-L	0.52	0.44	−0.078	−0.1434/−0.016	0.53	0.44	−0.085	−0.149/−0.024

*p < 0.05, **p < 0.01; PAS13 = Psychache Scale 13-item; PAS10 = Psychache Scale 10-item (Blandizzi et al., 2025); UP3, Unbearable Psychache 3-item (Pachkowski et al., 2019); PHQ-9, Patient Health Questionnaire; SHSS-C, Suicidal History Self-Rating Scale, last 12 months; SHSS-L, Suicidal History Self-Rating Scale Lifetime version; Zou’s CI, Zou’s (2007) confidence interval 95% confidence to compare pairs of correlations, two-tailed; Δr, difference between two pairs of correlations.

## 4 Discussion

Pachkowski et al. (2019) developed the Unbearable Psychache Scale (UP3), by selecting three items from the PAS13 (Holden et al., 2001), to specifically capture unbearable forms of psychache, theorized as the dimension of mental pain that directly triggers a suicidal crisis (Shneidman, 1996, 1998). Early evidence supported UP3’s strong internal consistency and incremental validity in predicting suicidal ideation, even when controlling for general psychache and other related constructs (Pachkowski et al., 2019). Conversely, Campos and Holden (2020) reported that, although the UP3 had high reliability and differentiated between individuals with and without a history of suicide attempts, it did not outperform PAS13 in predicting suicidal behavior, suggesting that unbearable psychache may be more strongly related to suicidal ideation than to the emergence of suicidal behaviors (Campos and Holden, 2020).

Results from both Study#1 and Study#2 confirmed a unidimensional structure of the UP3 and a good fit of a tau-equivalent model across samples. The three items capture a single latent dimension of unbearable psychache, with adequate internal consistency. The analyses also supported the assumption of monotonicity, with higher item scores corresponding to greater psychache, though invariant item ordering was weak to moderate, suggesting that the three items of the UP3 cannot be consistently ranked by difficulty across all participants.

Correlations with UP3 and PAS scales were high, although UP3 maintained high independent variance, denoting discriminant validity relative to broader measures of mental pain and supporting that UP3 captures only unbearable forms of psychache. Moreover, UP3 scores showed stronger associations with depression severity than PAS longer versions. Conversely, analyses examining associations with recent (SHSS-C) and lifetime (SHSS-L) suicide risk showed nuanced patterns: correlations with SHSS-C were indistinguishable between the UP3 and the PASs, also when partialling out depression severity; whereas correlations between the UP3 and the SHSS-L were slightly lower than those of the PAS longer versions. Hierarchical logistic regressions further showed that UP3 did not provide incremental validity over PAS13 for either recent or lifetime suicide risk, indicating that while the UP3 effectively captures acute intolerable psychache, PAS13 remains a more comprehensive predictor of overall suicidal risk. These patterns are not completely consistent with the theoretical model positing only unbearable psychache as a proximal risk factor for suicide (Shneidman, 1996, 1998; Pachkowski et al., 2019).

When examining the ability to discriminate between participants with different suicide risk, the ROC curves indicated that for recent suicidal ideation and behaviors (last 12 months) UP3 displayed fair to good accuracy, with AUCs broadly comparable to PAS13 and PAS10. Conversely, for lifetime suicide risk (except for the last 12 months), AUCs for UP3 were lower than those of PAS scales, with significant differences for lifetime suicidal ideation. For this measure, UP3’s AUC was also below the acceptable threshold (0.681). These results indicate that PAS scales are more effective at detecting lifetime suicide risk.

The examination of sensitivity, specificity, and PPV provided further insights. Notably, although AUCs were generally acceptable, sensitivity and specificity were suboptimal for both UP3 and PAS scales. Overall, the UP3 tended to maximize detection of true positives (high sensitivity) at the cost of false alarms (lower specificity), particularly for lifetime measures where sensitivity dropped markedly. In contrast, PAS13 and PAS10 exhibited more balanced profiles across both recent and lifetime risk indices.

Suboptimal indices of sensitivity and specificity were paired with low PPV indices. For recent suicidal ideation, the PPV of UP3 was 0.42 (sensitivity/specificity = 0.58/0.80), compared with 0.55 for PAS13 and 0.43 for PAS10 (sensitivity/specificity = ~0.56–0.64/0.79–0.88). For recent suicidal behaviors, the PPV of UP3 was 0.21 (sensitivity/specificity = 0.73/0.92), compared with 0.22–0.23 for the PAS scales (sensitivity/specificity = ~0.74–0.75/0.91–0.92).

These low PPVs highlight an important limitation for clinical application. Although UP3 and PAS scales can identify individuals at risk, a substantial proportion of positive cases may be false positives. This limitation is consistent with previous research. For instance, a meta-analysis by Carter et al. (2017) calculated the pooled estimates for PPV of clinical risk assessment instruments for subsequent suicidal and nonsuicidal self-injurious behaviors. The authors reported a pooled PPV of only 16% for any suicidal behaviors and any instruments, emphasizing the difficulty of accurately predicting suicidal outcomes even with validated measures (Carter et al., 2017). Accurate assessment of suicide risk is essential for clinicians, but predicting who will attempt or die by suicide remains challenging. Generally, the presence and severity of suicidal ideation have been used to predict future suicidal behaviors, but studies have indicated that many attempters deny suicidal ideation (Simpson et al., 2023; Berman, 2018). To improve risk prediction, other factors (e.g., hopelessness, mental pain, depression) and measures should be considered. Nevertheless, the low prevalence of suicidal behaviors in the general population limits the precision of any clinical scale, and even near-perfect instruments produce a high proportion of false positives (Carter and Spittal, 2018). Therefore, it is not recommended in clinical care to use only the UP3 or PASs scores for risk stratification, in line with the US Department of Veterans Affairs (2025), who assert that “there is insufficient evidence to recommend for or against the use of a specific tool or method to determine the level of risk.”

Taken together, these findings are broadly consistent with prior research showing that unbearable psychache and UP3 scores are strongly associated with acute suicide risk (Pachkowski et al., 2019). Although UP3 performance is similar to the PAS in detecting recent suicidal ideation and behavior, it shows slightly lower discriminative ability for lifetime suicide risk. In contrast, the broader psychache measures have shown stronger associations with lifetime suicide risk (DeLisle and Holden, 2009; Nahaliel et al., 2014; Campos and Holden, 2020; Blandizzi et al., 2025), reflecting their focus on enduring mental pain rather than its intolerable, proximal component. These findings can be interpreted in light of Shneidman’s model, which posits that suicide occurs when psychache reaches an unbearable threshold, representing the proximal driver of suicidal behavior (Shneidman, 1996, 1998; Troister and Holden, 2012). By targeting this “unbearable” component, the UP3 appears to be more sensitive to recent risk, while the PAS may better reflect lifetime risk, by capturing broader and more enduring manifestations of psychache. In other words, the UP3 may detect the immediate psychological tipping point that leads to suicidal thoughts or actions, while the PAS scales assess a more stable vulnerability over time. This distinction highlights an important theoretical implication: future research should examine whether focusing on unbearable psychache provides additional predictive utility for acute suicidal crises beyond general psychache measures.

Although interesting, our results should be interpreted in light of some limitations. First, the study relied exclusively on self-report measures, which may be influenced by social desirability, response bias, or participants’ willingness to disclose suicidal thoughts and behaviors. Second, both samples were drawn from the general population, with Study#2 based on a relatively small community sample. As a result, the prevalence of suicidal ideation and behavior is limited, potentially affecting statistical power and the stability of ROC estimates, especially for less frequent lifetime behaviors. These findings should therefore be interpreted cautiously, as they provide preliminary insights rather than definitive conclusions. Future studies with larger samples are needed to confirm the observed patterns and more accurately assess the performance of UP3 and PAS scales in detecting suicide risk. Third, both Study #1 and Study #2 samples were predominantly composed of female participants. Although the MIMIC model indicated a significant effect of sex, with females reporting higher UP3 scores than males, the gender imbalance may still limit the generalizability of our findings. Future studies should aim to include more balanced samples to evaluate potential gender-related differences in the performance of the UP3 and PAS scales. Fourth, all data were cross-sectional, limiting the ability to establish temporal or causal relationships between unbearable psychache and suicidal outcomes. This also constrains the ability to determine whether UP3 can predict future suicidal behaviors compared with the PAS. Longitudinal studies are needed to evaluate the predictive utility of UP3 over time. Fifth, the adoption of only three items to operationalize the construct of “unbearable psychache” entails inherent limitations. Using so few indicators increases the risk of construct underrepresentation, as the full complexity of unbearable psychache may not be fully captured. Furthermore, the adoption of a tau-equivalent model with only three items remains statistically constrained. Although the Mokken Scale Analysis provides convergent evidence that the items reliably measure a single latent construct, the assumption that all items contribute equally to the latent factor should be interpreted with caution. Moreover, the psychometric properties and discriminative validity of the UP3 should be tested in high-risk clinical populations (e.g., psychiatric inpatients, individuals with recent suicide attempts) to assess generalizability and clinical utility beyond non-clinical settings. Finally, the present study did not examine potential moderating variables such as age, gender, psychiatric diagnosis, or comorbid conditions, which could influence the relationship between unbearable psychache and suicide risk. Future research should explore these moderators to better understand for whom and under which conditions the UP3 is most predictive.

Despite these limitations, the present study also has several strengths. It is the first study, to our knowledge, to examine the factor structure and scalability of the UP3 using both CFA and MSA. This improves factorial validity, reliability, and enhances criterion validity, particularly when using summed items in analyses (Czerwiński and Atroszko, 2023). Furthermore, our sample size in Study #1 fully meets and exceeds the criteria suggested by Straat et al. (2014) to ensure reliable partitioning and scalability results (Watson et al., 2018). These methodological strengths add robustness to our findings and contribute to the growing literature on the measurement of unbearable psychache.

Overall, the present study highlights limit and strengths of the UP3. Its brevity and ease of administration make it particularly suitable for use in clinical settings or large-scale screenings, where time constraints and patient burden can limit the feasibility of longer assessments. By specifically targeting the unbearable dimension of psychache, the UP3 offers a concise yet theoretically grounded tool that could help clinicians and researchers quickly identify individuals experiencing acute mental pain associated with heightened suicide risk. Nevertheless, we should point out that no clinical instruments can assess suicide potential with acceptable precision, and the UP3 should be used in conjunction with other assessment tools as a part of a broader evaluation of suicide risk.

## Data Availability

The original contributions presented in the study are included in the article, further inquiries can be directed to the corresponding authors.
